# A case report of glucose transporter 1 deficiency syndrome with growth hormone deficiency diagnosed before starting ketogenic diet

**DOI:** 10.1186/s13052-020-00888-3

**Published:** 2020-08-26

**Authors:** Gianluca Tornese, Giuseppa Patti, Maria Chiara Pellegrin, Paola Costa, Flavio Faletra, Elena Faleschini, Egidio Barbi

**Affiliations:** 1 Institute for Maternal and Child Health IRCCS “Burlo Garofolo”, Via dell’Istria 65/1, 34137 Trieste, Italy; 2 Department of Neuroscience, Rehabilitation, Ophtalmology, Genetics, Maternal and Child Health, University of Genova, Genova, Italy; 3 University of Trieste, Trieste, Italy

**Keywords:** Short stature, Seizure, SLC2A1, Hypoglycemia, Ketogenic diet, Case report

## Abstract

**Background:**

Growth failure and growth hormone deficiency (GHD) have been reported as one accessory feature of GLUT1 deficiency syndrome (GLUT1DS), considered so far as a long-term adverse effects of ketogenic diet which is used to treat this condition**.**

**Case presentation:**

We report the case of a 10-year-old Caucasian boy referred for short stature (height − 2.56 SDS) and delayed growth (growth velocity − 4.33 SDS) who was diagnosed with GHD and started treatment with recombinant human growth hormone (rhGH). Because of his history of seizures with infantile onset, deceleration of head growth with microcephaly, ataxia, and moderate intellectual disability, a lumbar puncture was performed, which revealed a low CSF glucose concentration with a very low CSF-to-blood glucose ratio (< 0.4), and genetic tests detected a *SLC2A1* gene exon 1 deletion confirming a diagnosis of GLUT1DS. Ketogenic diet was started. After 5.5 years of rhGH treatment his height was normalized (− 1.15 SDS). No side effects were reported during treatment, particularly on glycemic metabolism.

**Conclusions:**

This is the first case of GHD in a Caucasian boy with GLUT1DS diagnosed before starting ketogenic diet, with a good response to rhGH treatment and absence of side effects. We speculate that GHD may represent a poorly recognized clinical feature of GLUT1DS rather than a complication due to ketogenic diet. Under-diagnosis may derive from the fact that growth failure is usually ascribed to ketogenic diet and therefore not further investigated. Pediatric neurologists need to be alerted to the possible presence of GHD in patients with GLUT1DS with slow growth, while pediatric endocrinologist need to refer GHD patients with additional features (motor and cognitive developmental delay, seizures with infantile onset, deceleration of head growth with acquired microcephaly, movement disorder with ataxia, dystonia, and spasticity) that may suggest GLUT1DS.

## Background

Glucose transporter 1 deficiency syndrome (GLUT1DS) is caused by heterozygous, mostly de novo, mutations in the *SLC2A1* gene encoding the glucose transporter 1 (GLUT1). Mutations in this gene impair GLUT1-mediated glucose transport across the blood brain barrier, leading to cerebral energy deficiency. The clinical phenotype is characterized by the variable association of motor and cognitive developmental delay, seizures with infantile onset, deceleration of head growth, often resulting in acquired microcephaly, and a movement disorder with ataxia, dystonia, and spasticity [1]. During the last years the classic phenotype has expanded, and atypical phenotypes have been reported, with the absence of some characteristics (seizures, movement disorders or decrease of head circumference) or the presence of other features (episodic choreiform movements, infants with reversible hypoglycorrhachia) [2]. The diagnostic hallmark is a reduced glucose concentration in the cerebral spinal fluid (CSF) with a lowered CSF-to-blood glucose ratio. GLUT1DS is effectively treatable with ketogenic diet, which provides ketones as an alternative fuel for the brain [3]. While it is known that ketogenic diet may cause growth retardation [4], growth hormone deficiency (GHD) may represent another possible cause of growth failure in children with GLUT1DS [5, 6]. We report the first case of GHD in a Caucasian boy with GLUT1DS diagnosed before starting ketogenic diet, with a good response to recombinant human growth hormone (rhGH).

## Case presentation

**Fig. 1:**
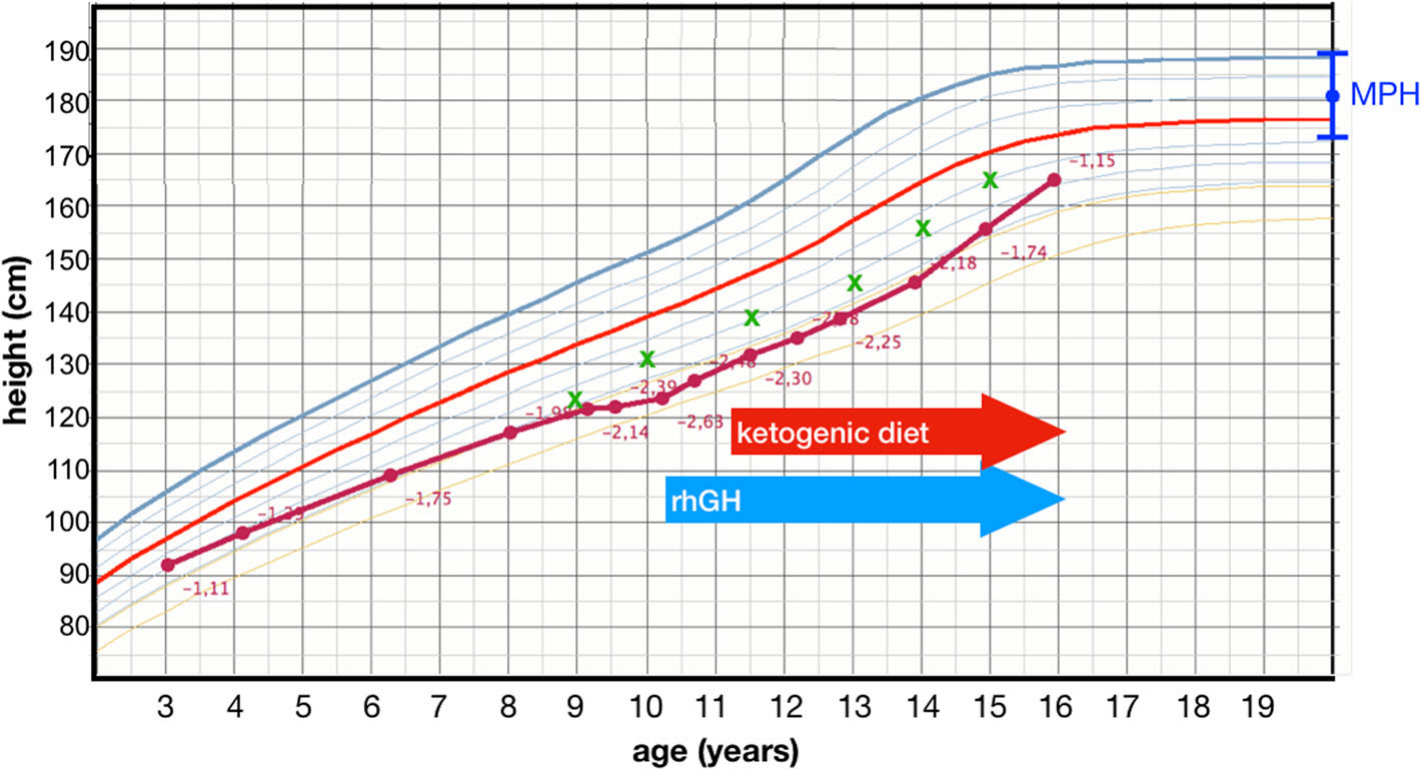
Growth chart of the patient from 3 to 16 years of age (green crosses: height referred to bone age; MPH: mid-parental height; rhGH: recombinant human growth hormone)

A 10-year-old Caucasian boy was referred for evaluation of short stature and growth delay. His height was 123.8 cm (− 2.63 SDS), his weight was 22.5 kg (− 2.59 SDS), BMI was 14.68 kg/m^2^ (− 1.71 SDS) [7], head circumference was 50 cm (− 2.30 SDS) [8]. Growth velocity was 2.1 cm/year in the previous year (− 4.37 SDS) [9] *(*Fig. 1*)*. His genitals were prepuberal (Tanner I). At physical examination, he had mild facial dysmorphism (epicanthus, telecanthus, hypertelorism) and ataxia.

He was born at 38 weeks of gestation via an uncomplicated vaginal delivery, with an Apgar score of 9–10 at 1 and 5 min, respectively. Birth weight was 2.674 kg (− 1.29 SDS); birth length was 47 cm (− 1.29 SDS); head circumference was 32 cm (− 1.75 SDS) [10].

From the first months of life he showed development delay, with feeding difficulties and first steps at the age of 17 months. At the age of 11 months he developed seizures for which he was treated with ethosuccimide with good control. From the age of 3 years, he developed ataxia; at that time his height was 92 cm (− 1.11 SDS) [7] *(*Fig. 1*)* and head circumference 47.5 cm (− 1.28 SDS) [8]; a brain magnetic resonance imaging (MRI) was performed, which showed a diffuse hyperintensity of white matter signal on T2-weighed images. Audiometry, eye and cardiac examinations were normal. At the age of 7, he was diagnosed with a moderate intellectual disability (IQ 40) at Wechsler Intelligence Scale for Children, fourth edition (WISC-IV) [11]. No final diagnosis was reached.

There was no familial history of short stature, with a mid-parental height (MPH) of 181 cm (0.73 SDS) [7].

Two GH provocative tests (arginine and clonidine, without sexual hormones priming) were performed, showing GHD (GH peak: 7.5 ng/ml on the first test, 6.4 ng/ml on the second test), with insulin-like growth factor 1 (IGF-1) in the lower range (− 1.32 SDS). Bone age was 9 years (Greulich&Pyle) with a predicted adult height of 163.5 cm (− 2.10 SDS) [12], below the MPH range. Other common causes of delayed growth, such as hypothyroidism, chronic conditions, malnutrition, Cushing disease, celiac disease, were ruled out by laboratory tests. An additional brain MRI was performed, which again revealed a non-specific high T2 signal of white matter, while pituitary gland was normal for size and there were no mass lesions. The patient was started on rhGH replacement therapy at a dose of 26 mcg/kg/day [13].

Due to his neurological history (seizures with infantile onset, deceleration of head growth with microcephaly, ataxia, and moderate intellectual disability), GLUT1DS was suspected. A lumbar puncture was performed, revealing a low CSF glucose concentration (hypoglycorrhachia) with a very low CSF-to-blood glucose ratio (< 0.4). Genetic tests were performed, which detected a *SLC2A1* gene exon 1 deletion. A diagnosis of GLUT1DS was established [1] and ketogenic diet was started one year after the beginning of rhGH treatment. During that time the boy increased his height by 6.3 cm (0.27 SDS).

Treatment with rhGH was continued (up to a dose of 32 mcg/kg/day), with 6-monthly follow-ups, with a good compliance to treatment. He started puberty at the age of 13.5 years. At 16 years of age, after 5.5 years of therapy, his height was 165.1 cm (− 1.15 SDS) on a rhGH dose of 30 mcg/kg/day, with a growth velocity of 9.5 cm/year, at a Tanner stage IV, IGF-1 in the normal range (384 ng/ml, − 0.54 SDS) and bone age still 1 year delayed compared to chronological age *(*Fig. 1*)* with predicted adult height of 172.5 cm (− 0.65 SDS) [12], in the low range of MPH. No side effects were reported during treatment, particularly on glycemic metabolism. Patient and parents were highly satisfied with the outcome of rhGH treatment.

## Discussion and conclusions

We report the first case of GHD in a Caucasian boy with GLUT1DS, diagnosed before starting ketogenic diet and successfully treated with rhGH replacement therapy with absence of side effects.

The co-occurrence of GHD and GLUT1DS has been reported only in a few Japanese children so far. In 2005 Etani et al. reported a small case series with a possible association, in children (mean age 6.25 years) with a mean height of − 2.7 SDS [5]. Nakagama et al. described a boy aged 12 years and 7 months with GLUT1DS complicated with severe GHD (height at diagnosis − 3.6 SDS, growth velocity 1.7 cm/year, peak at GH stimulation test 1.92 ng/ml, low IGF-1, normal pituitary at MRI) successfully treated with GH replacement therapy, at a dose of 25 mcg/kg/day [6]. However, the diagnosis of GHD was established when the boy was already been diagnosed with GLUT1DS and treated with a ketogenic diet for more than 4 years, therefore the role of ketogenic diet on GHD could not be completely ruled out at that time. When ketogenic diet was started, the child did not present short stature (height − 1.9 SDS), however slow growth was already present (height was ∼ − 1 SDS until the age of 5 years); moreover, there was a clinical improvement following rhGH replacement (growth velocity of 7.5 cm/year at first year and 4.3 cm/year at second year), suggesting that growth failure was due to GHD and that it was present even before the starting of ketogenic diet.

Differences in GH peak and IGF-1 levels between our case (mild GHD) and the one described by Nakagama et al. (severe GHD) may be ascribed to the additional negative effect of ketogenic diet on GH secretion, due to reduced ghrelin availability [14]. Notably, in our case the diagnosis of GHD was established before the diagnosis of GLUT1DS, completely excluding the confounding role of ketogenic diet. Moreover, treatment was effective, leading to an increase of nearly 1 SDS over 5 years of treatment.

No data are available so far on the persistence of GHD after reaching adult height in GLUT1DS patients. Based on GH peaks at stimulation tests in our patient, we may speculate that it could be a transient GHD, not requiring further treatment in adulthood, [15] although additional studies are needed. Particularly, it is not clear whether we can rely on GH stimulation tests or retesting while ketogenic diet is still ongoing [14].

A possible explanation for pathophysiological direct link between GH deficiency and GLUT1DS is that GH has a role in the counter-regulatory hormonal response to hypoglycemia, and it is well-known that this counter-regulatory response is blunted in patients with recurrent severe hypoglycemia. Neural cells of GLUT1DS patients are, in fact, chronically deprived of glucose supply, a situation that could physiologically resemble the effect of repeated severe hypoglycemia. Thus, impaired counter-regulatory hormonal response to hypoglycemia could cause GHD in children with GLUT1DS [6]. Interestingly, acquired GHD in GLUT1DS may also represent an adaptation phenomenon. GH/IGF1 deficient animals exhibit decreased glucose metabolism in many brain regions, which is independent of GLUT1 activity [16]. GLUT1DS patients, whose neural cells are chronically deprived of glucose supply, may also have a reduced GH/IGF1 axis activity as a negative feedback, and develop GHD as a result [6]. Nevertheless, normal MRI images sustain that GHD is not related to a reduced size of pituitary gland.

We therefore speculate that GHD may represent a poorly recognized clinical feature of GLUT1DS rather than a complication of ketogenic diet and could be not properly diagnosed before: that under-diagnosis could derive from the fact that growth failure may be ascribed to ketogenic diet only and therefore not further investigated. In the previously described cases, GLUT1DS was diagnosed by neurologists, ketogenic diet was started and children were reported to endocrinologists only when heights were close to − 3 SDS. However, there are no comprehensive reports on growth and GH secretion in GLUT1DS patients, therefore the true prevalence of slow growth and GHD complicating GLUT1DS is not known.

Since neurological symptoms are those who lead to the diagnosis of GLUT1DS, pediatric neurologists need to be alerted to the possible presence of GHD in patients with GLUT1DS and follow their growth. At the same time, pediatric endocrinologists need to refer GHD patients with additional features (motor and cognitive developmental delay, seizures with infantile onset, deceleration of head growth with acquired microcephaly, movement disorder with ataxia, dystonia, and spasticity) that may suggest GLUT1DS. Additional studies are needed to study growth and GH secretion in GLUT1DS patients and to verify the prevalence of GHD associated to GLUT1DS.

## Data Availability

Data sharing not applicable to this article as no datasets were generated or analysed during the current study.

## Abbreviations

CSF: cerebral spinal fluid
GHD: growth hormone deficiency
GLUT1: glucose transporter 1
GLUT1DS: glucose transporter 1 deficiency syndrome
MPH: mid-parental height
MRI: magnetic resonance imaging
rhGH: recombinant human growth hormone
SDS: standard deviation score
